# Multi-wavelength structured light based on metasurfaces for 3D imaging

**DOI:** 10.1515/nanoph-2023-0885

**Published:** 2024-02-07

**Authors:** Baiying Lyu, Chen Chen, Jian Wang, Chang Li, Wei Zhang, Yuxiang Feng, Fei Dong, BaoShun Zhang, Zhongming Zeng, Yiqun Wang, Dongmin Wu

**Affiliations:** School of Nano-Tech and Nano-Bionics, University of Science and Technology of China, Hefei 230026, China; Nanofabrication Facility, Suzhou Institute of Nano-Tech and Nano-Bionics, Chinese Academy of Sciences, Suzhou 215123, China; Beijing Aerospace Institute for Metrology and Measurement Technology, Beijing 100076, China

**Keywords:** metasurface, structured light, multi-wavelength, three-dimensional imaging, resolution

## Abstract

Structured light projection provides a promising approach to achieving fast and non-contact three-dimensional (3D) imaging. The resolution is a crucial index that represents security and accuracy in applications such as face recognition and robot vision. It depends on the density of dots in the projection. However, further improving the density of dots in the current system must be at the cost of speed or volume. Here, an all-dielectric ultra-thin metasurface is designed and fabricated to project a multi-wavelength dot array. The density of dots is improved because projected dots with different wavelengths fill the gaps with each other. The experimental results demonstrate that the multi-wavelength projection improves the resolution of 3D imaging. Furthermore, the multi-wavelength system is beneficial to measuring a surface with varying colors. The approach has the potential to achieve a new generation of high-resolution systems for tiny fluctuations and colorful 3D imaging in dark environments.

## 1 Introduction

Structured light (SL) projection has been widely used in three-dimensional (3D) imaging [1] applications such as face recognition [2], [3], [4], robot vision [5], [6], and autonomous driving [7], [8], with advantages in high speed and accuracy. Resolution is a crucial index for security and accuracy [9] in 3D measurement, which is determined by the density of dots in the projection. However, the maximum dot density is limited because adjacent dots with identical wavelengths must have gaps to be distinguished [9]. The limitation exists in most current SL systems, which makes a challenge to detect tiny fluctuations on the surface. To break the limitation, varied wavelengths can be introduced into the SL projector [10], [11]. The dots diffracted by different wavelengths interlace, filling the gaps at other wavelengths. These dots, characterized by different wavelengths, will be separated by a color camera, ensuring they do not affect each other in 3D imaging. Diffractive optical elements (DOEs) are commonly employed to generate SL projection [12], [13] on mobile devices. However, DOEs modulate the phase using the varied heights of structures, which results in intrinsic dispersion. The dispersion leads to a decrease in the signal-to-noise ratio, rendering some dots unrecognizable and causing a reduction in accuracy in the 3D reconstruction results. Therefore, an optical element that can project a high-performance multi-wavelength dot array is needed.

As a type of novel ultra-thin optical element, metasurfaces [14] can precisely control the phase [15], [16] and dispersion [17], [18], [19] by modulating the subwavelength structures [20], [21]. The remarkable properties make metasurfaces highly promising for various optics and photonics applications such as full-color imaging [22], [23], holographic [24], [25], [26], polarizing elements [27], and beam shaping [28], [29]. Recently, metasurface-based SL projections [30], [31], [32] have successfully accomplished high-quality single-shot 3D imaging [8], [33] with a single-wavelength system [34]. The ability of the metasurface to modulate identical phase distributions makes it well suited for using in multi-wavelength SL systems. Here, we propose Pancharatnam–Berry (PB) phase metasurfaces to project a high-density array of dots with multiple wavelengths. The dot number of the projection is triple the number of single-wavelength methods in the same area. Dots with different colors interlace with one another and are distributed without affecting one another. The reconstructed result shows finer surface details compared to the single-wavelength method, demonstrating an improved resolution with the multi-wavelength approach. Furthermore, the method can solve the problem that a single-wavelength projection may be disturbed by objects with specific colors due to low reflectivity. The adoption of the multi-wavelength approach has the potential to achieve high-resolution, colorful 3D imaging in dark environments and enable a new generation of systems for more applications.

## 2 Design and characterize of the multi-wavelength diffractive metasurface

**Figure 1:**
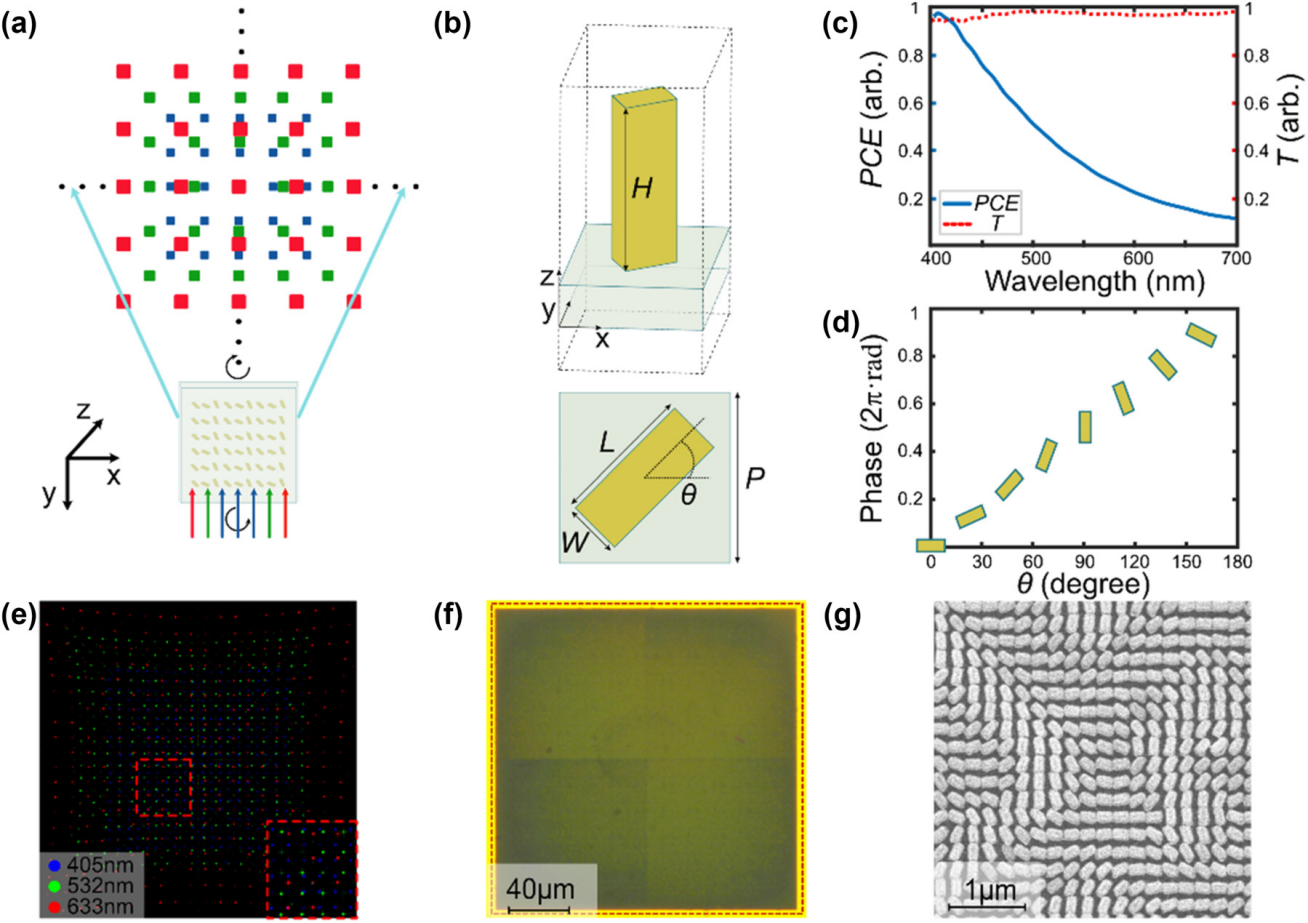
Design and fabrication of the metasurface. (a) Schematic of the principle of projecting a multi-wavelength SL dot array by metasurface. (b) Side view and top view of a unit cell. In the unit cell, *H* = 500 nm, *P* = 200 nm, *L* = 165 nm, and *W* = 65 nm. (c) Simulation results of *PCE* and *T* with respect to the wavelength. (d) Simulation results of the PB phase, which is twice the rotation angle *θ*. (e) Simulation of the multi-wavelength diffraction patterns calculated using Rayleigh–Sommerfeld diffraction integral. (f) Optical image of the fabricated metasurface obtained using optical microscopy. (g) SEM image of the fabricated metasurface obtained using scanning electron microscopy.

As shown in Figure 1(a), the metasurface is designed to modulate identical phase distributions at three incident wavelengths of 405 nm, 532 nm, and 633 nm. The coordinates of each diffraction order increase with the wavelength, allowing dots to interlace with each other. For higher transmission, the cell of the metasurface consists of TiO_2_ nanofin on the SiO_2_ substrate, which demonstrates a near-zero absorption in the visible spectrum. Figure 1(b) shows the schematic diagram of the cell of the metasurface. The dimensional parameters of the nanofin are as follows: size *P*, length *L*, width *W*, height *H*, and angle *θ*. PB phase *φ* for each cell is only determined by the rotation angle *θ* for nanofin according to the relationship of *φ* = ±2*θ*. The rotation angle can be almost continuous and arbitrary without adding difficulty to the fabrication. The transmission efficiency (*T*) and polarization conversion efficiency (*PCE*) are determined by the other dimensional parameters. Here, the nanofin is designed as a half-wave plate to convert light from the left-handed circularly polarized (LCP) to the right-handed circularly polarized (RCP) for maximal *T* and *PCE*. The parameters of nanofins, including *L*, *W*, *P*, and *H*, are optimized at 405 nm (see Supplementary Material Note 1). The fabrication ability is also considered in the optimization for consistency before and after processing. Supplementary Material Figure S1 shows the simulation results. For the optimized nanofin with *L* = 165 nm and *W* = 65 nm, *T* is as high as 94.63 %, and *PCE* is as high as 94.76 % at 405 nm. The *PCE* and *T* at visible light are shown in the blue curve and red dashed line of Figure 1(c), respectively. At the wavelengths of 532 nm and 633 nm, *T* is 97.52 % and 96.57 %, and *PCE* is 41.93 % and 22.60 %, respectively. As shown in Figure 1(d), the PB phase is twice the rotation angle *θ* regardless of wavelength.

In this work, the target projection consists of 1200 dots from three different wavelengths, with the entire area of the dot array designed as 50 mm × 50 mm. There is a 20 × 20 dot array with a design wavelength of 405 nm. The size of the dots is 0.2 mm × 0.2 mm. The gap between two adjacent dots is approximately 2.19 mm. The distance between the metasurface and the diffraction plane is 100 mm. Supplementary Material Figure S2 shows the target dot array.

The phase distribution of the metasurface is calculated using scalar diffraction theory and Gerchberg–Saxton (G-S) algorithm [35]. During the iteration process, the light intensity utilization in the field (*LIUF*) is employed to validate the diffraction simulation result (Details in Supplementary Material Figure S3). The *LIUF* is calculated as follows:

$$LIUF=\frac{\sum I_{\mathrm{dot}}}{\sum I_{\mathrm{field}}},$$

where *I*_*dot*_ is the light intensity for each dot, and *I*_*field*_ is the light intensity for the entire field of the diffracted pattern. A higher *LIUF* implies that stray light has a weak effect on the projection. For the 405-nm incident light, the final *LIUF* exceeds 73.34 % without the zero-order diffraction. For the 532-nm and 633-nm incident light, *LIUF* is 70.64 % and 66.64 %, respectively (Details about zero-order diffraction are provided in Supplementary Material Note 3). In addition, the uniformity of the intensity distribution (*RMSE*) is used to validate the quality of the dot array. The *RMSE* is calculated as follows:

$$RMSE=\sqrt{\frac{{\underset{k\in dot}{\sum }\left[\frac{I_{k}-\bar{I}}{\bar{I}}\right]}^{2}}{n-1}},$$

where *dot* represents all dots in the diffraction pattern, *I*_*k*_ is the intensity of the *k*th dot, $\bar{I}$ is the average intensity of all dots, and *n* is the number of dots. A low *RMSE* indicates that the dots have similar intensities, i.e., a good performance. For the three incident wavelengths, the *RMSE* is 18.82 %, 66.54 %, and 68.31 %, respectively. The *RMSE* increases when the incident wavelength moves away from the design wavelength. Supplementary Material Figure S3 shows the *RMSE* with the number of iterations. Supplementary Material Figure S5 shows the iterated phase distribution. Based on the phase distribution, the diffraction light field under three wavelengths is simulated using Rayleigh–Sommerfeld diffraction integral formula, as shown in Figure 1(e). The fabrication details of the metasurfaces are provided in Supplementary Material Note 5. Figure 1(f) and (g) show the optical image and scanning electron microscopy (SEM) image of nanofins.

**Figure 2:**
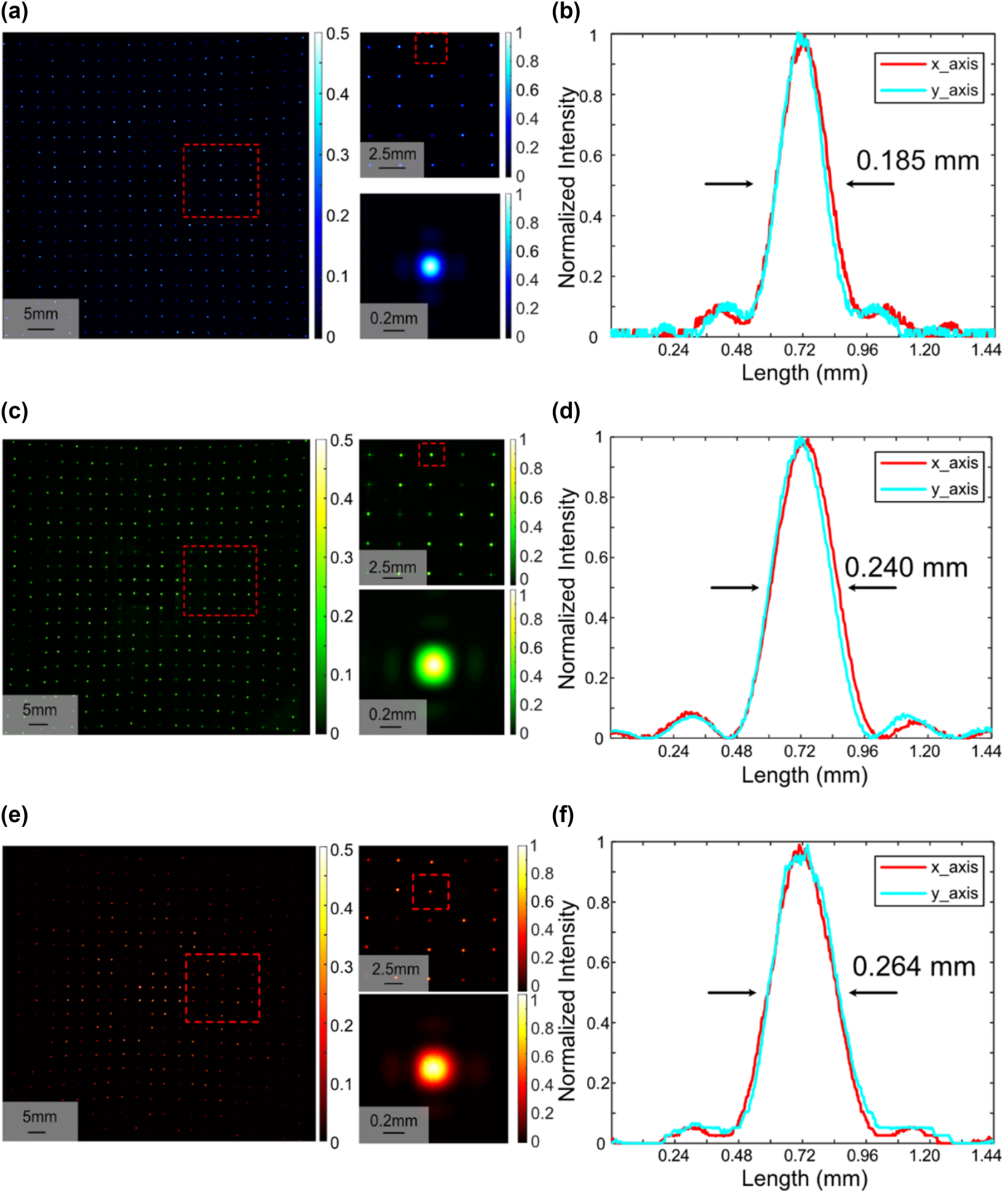
Characterization of the fabricated metasurface. (a), (c), (e) Diffraction pattern obtained by the camera. The dot array has 400 spots with 20 points per row and 20 points per column at wavelengths of 405 nm, 532 nm, and 633 nm. The red dotted boxes show the positions of the zoomed views within the 20 × 20 dot array and 5 × 5 dot array. (b), (d), (f) Relative intensity in the *x*-axis and *y*-axis at the center of the single dot in (a),(c), (e). FWHMs of the dots are labeled on the plots.

To characterize the optical performance of the proposed metasurface, a measurement setup is built to image the diffraction pattern (Details are provided in Supplementary Material Note 6). Supplementary Material Figure S6 schematically illustrates the measurement setup. To obtain the complete diffraction pattern, we took an image of every six dots with two rows and three columns. Adjacent images have a common row or column of identical diffraction order to ensure that the relative position of each image is correct when we stitch them together into an entire image. Figure 2(a) shows the complete diffraction pattern of the 405-nm case, which exhibits a slight pincushion distortion similar to the simulation (Details about light intensity distribution are provided in Supplementary Material Note 7). The diffraction pattern nearly conforms to the simulation results (Details about diffraction angles are provided in Supplementary Material Note 8). For the obtained diffraction pattern, *LIUF* is 38.75 % without calculating zero-order diffraction. Figure 2(b) shows the normalized intensity distributions for a dot along the *x*-axis and *y*-axis. The *RMSE* of the dot array is 38.81 %. For the results at 532 nm and 633 nm, as shown in Figure 2(c) and (e), *LIUF* is measured as 44.97 % and 42.46 % without zero-order diffraction, respectively. Figure 2(d) and (f) illustrate the relative intensity profiles along the *x*-axis and *y*-axis of a single dot. The measured *RMSE*s are 57.24 % and 64.49 %, respectively. In addition, a polarizer is added between the metasurface and the camera to measure the polarization of the diffraction pattern (Details about the method of measuring the polarization are provided in Supplementary Material Note 9). Supplementary Material Figure S9 shows the polar map of the dots.

## 3 Performance of the metasurface-based multi-wavelength 3D imaging

**Figure 3:**
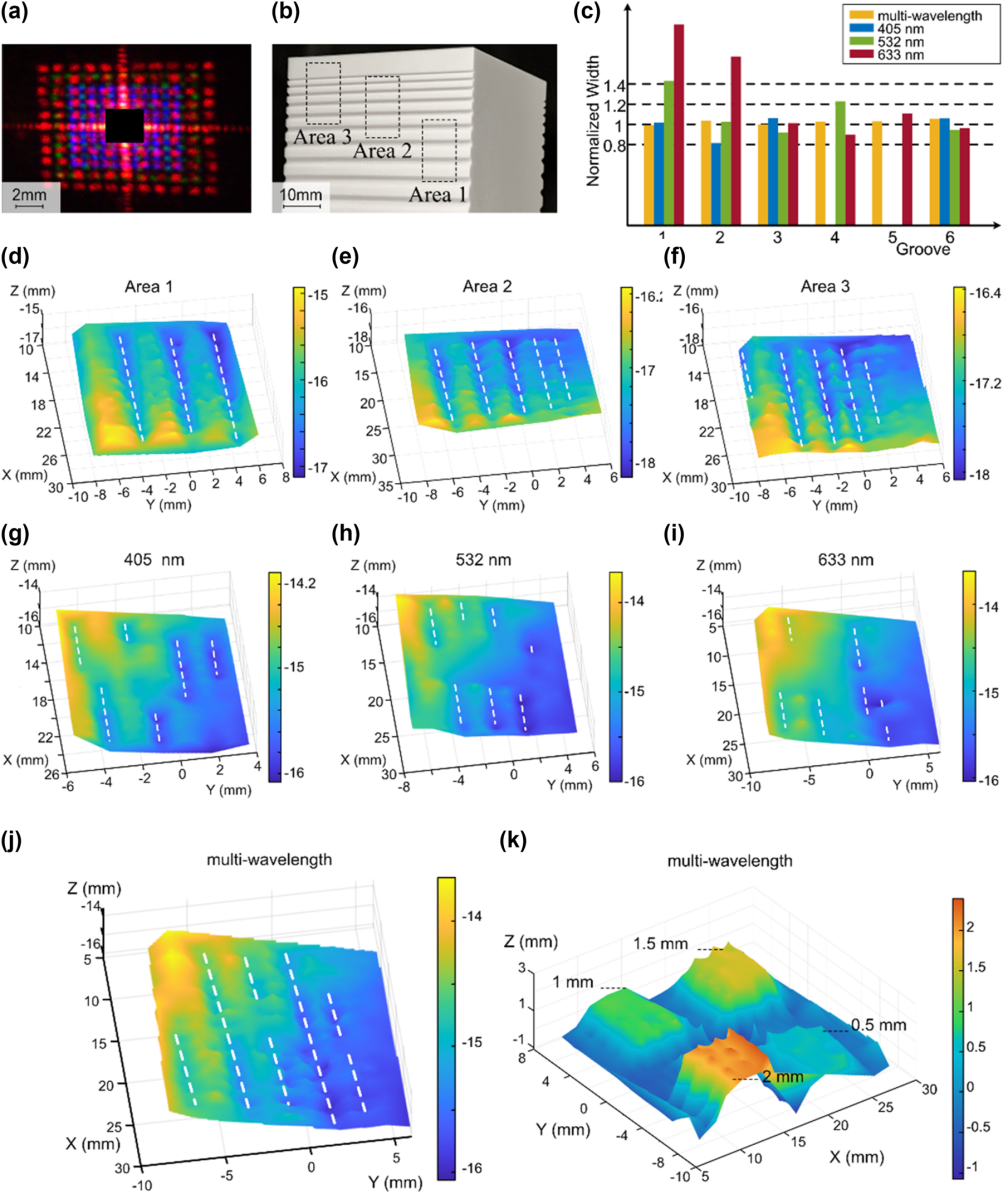
Surface maps obtained by metasurface-based multi-wavelength 3D imaging. (a) Image of the multi-wavelength dot array on the reference plane. (b) Image of three areas on the measured sample 1. (c) Average widths of the grooves, which are calculated by multi-wavelength imaging results and three single-wavelength imaging results. The average width is normalized to theoretical values. (d)–(f) Image of the surfaces of Areas 1–3 obtained by interpolating the point cloud of the multi-wavelength. The white dash lines represent the grooves. (g)–(i) Surface obtained by interpolating the point clouds of the wavelengths 405 nm, 532 nm, and 633 nm. The white dash lines represent the grooves. (j) Surface obtained by interpolating the point clouds of the multi-wavelength case. The white dash lines represent the grooves. (k) Image of the surface of step, which is obtained by interpolating the point clouds of the multi-wavelength case.

To validate the practical effectiveness of the multi-wavelength SL projection in 3D imaging, a metasurface to project an 11 × 11 dot array at three wavelengths is designed and fabricated. The projection is filled with 363 dots of three wavelengths and is approximately 10 mm × 10 mm at the work distance of 150 mm. The size of the dot is 0.8 mm × 0.8 mm. Within the range of the 405-nm diffraction pattern, the gaps between two adjacent dots of one wavelength are filled by the dots of other wavelengths. Figure 3(a) shows the diffraction pattern at the reference plane. A monocular vision system is built to measure the depth of a 3D-printed sample. The processed metasurface projects a dot array onto the surface, and a camera captures the backscattered light. The camera has been corrected using a checkerboard. Supplementary Material Figure S10 shows the checkerboard images.

The format of images obtained by the camera is RAW in the process of obtaining the pixel coordinates of each dot. Pixel coordinates refer to the corresponding pixel positions of the dots in the images. Ten images of the reference plane and the measured sample are captured and averaged to decrease noise. Then, the dot array of three wavelengths is separated into three patterns through the RGB channels. The pixel coordinates of each dot on the reference plane are calculated and extracted from the three obtained patterns. Afterward, a similar process is repeated to extract pixel coordinates from the images of the measured sample. Because of the varying depth of the measured sample, the order of pixel coordinates extracted from the sample may be different from the reference plane. A correspondence of the order of each projected dot is established between the measured sample and the reference plane. The spatial coordinates of each dot on the measured sample are calculated by the differences of the same channel between the sample and reference plane based on the triangulation principle, as shown in Supplementary Material Figure S11. The 3D point cloud of the measured sample is reconstructed by the spatial coordinates. And an interpolation process is executed on the point cloud to reestablish the surface of the measured sample. Details about the monocular vision depth calculation method are provided in Supplementary Material Note 11.

A measured sample 1 with grooves of varied widths is made using 3D printing, as shown in Figure 3(b). The widths of the grooves are decreased from 2.4 mm to 0.8 mm with a step of 0.2 mm. Three areas on the surface are measured using multi-wavelength projections, as shown in Figure 3(b). The calculated spatial coordinates are presented in the form of point clouds, as shown in Supplementary Material Figure S14. Figure 3(d)–(f) show the three reconstructed surfaces for three measured areas. Five cross-sections are chosen for each surface, and average widths of grooves are calculated using these cross-sections. Figure 3(c) shows the normalization average widths of the grooves. The multi-wavelength imaging results are closer to the theoretical width than the three single-wavelength imaging results. Here, some single-wavelength imaging data is missed due to the short-wavelength projection cannot fully cover the same groove with a long-wavelength in the same single-shot. Supplementary Material Table S4 shows the detailed width data. Another measured sample 2 with steps of varied depths is made using 3D printing, as shown in Supplementary Material Figure S15. The height difference between each step is 0.5 mm. As shown in Figure 3(k), the average heights of the four steps are 0.5964 mm, 0.9098 mm, 1.4575 mm, and 2.0125 mm, respectively. The depth resolution of the metasurface-based multi-wavelength system is approximately 0.5 mm (Details about the depth data are provided in Supplementary Material Note 13).

The multi-wavelength imaging method improves the resolution because the multi-wavelength projection dots are staggered with one another, fill the gaps between single-wavelength projection dots, and reduce the loss of 3D information. Comparing the reconstructed surfaces by multi-wavelength and three single-wavelength in Area 3, we find that the five reconstructed grooves from the multi-wavelength imaging result are complete, whereas all three single-wavelength results are incomplete. Figure 3(g)–(j) show the results of the multi-wavelength surface and three single-wavelength surfaces. The lateral resolution exhibits noticeable improvements with the multi-wavelength method. Supplementary Material Figure S17 shows the results of the three single-wavelength point clouds.

**Figure 4:**
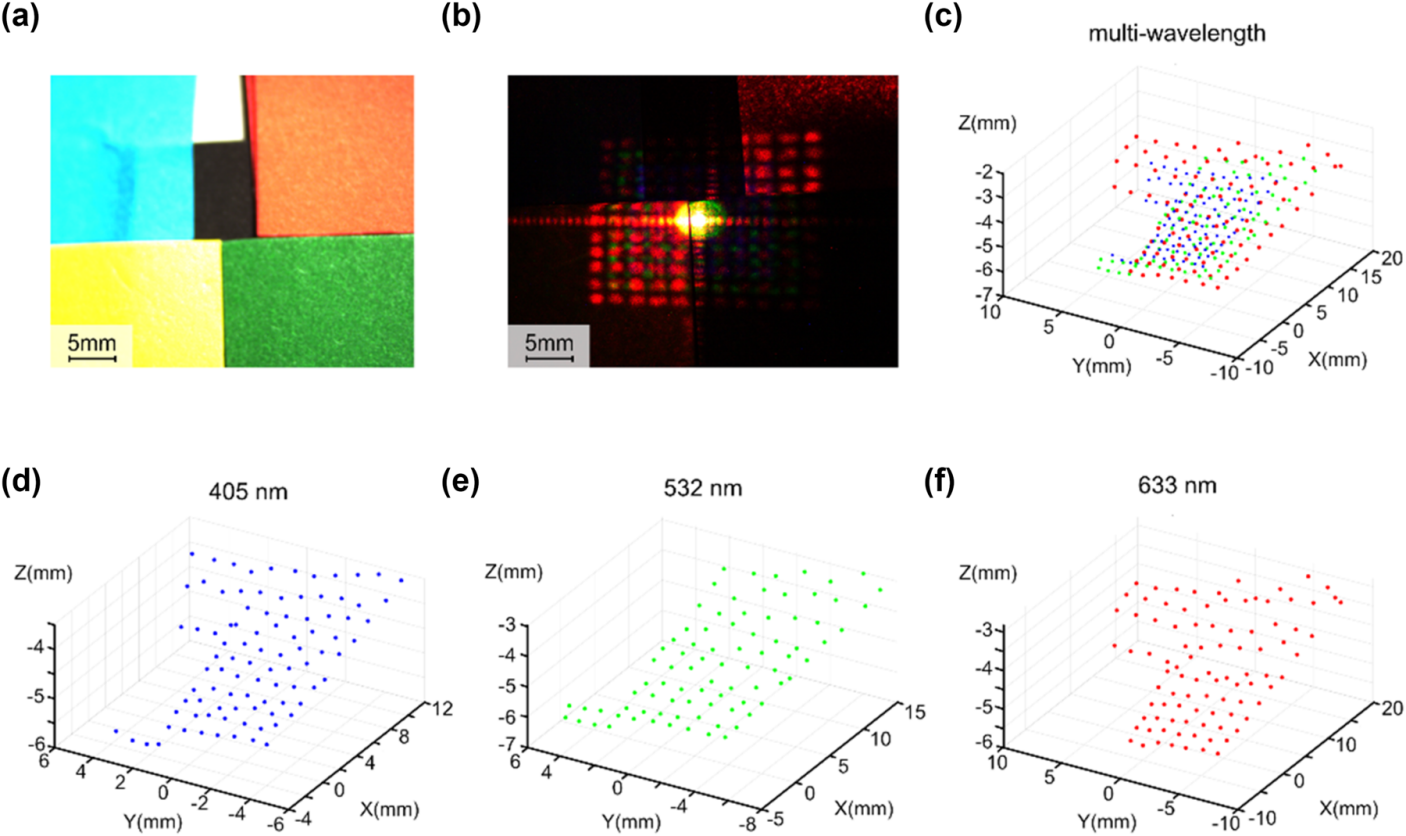
Metasurface-based multi-wavelength 3D imaging of five different colors of cardboard. (a) Image of the multi-wavelength dot array on five different colors of cardboard in natural light. (b) Image of the multi-wavelength dot array on five different colors of cardboard in measure environment. (c) Point cloud distribution map of the measured sample obtained by the multi-wavelength. (d)–(f) The point cloud distribution map is obtained by 3D imaging of the wavelengths 405 nm, 532 nm, and 633 nm.

Furthermore, the multi-wavelength method can resist the interference of varied colors of the measured sample. The metasurface-based multi-wavelength SL is employed to measure cardboards with five different colors: blue, black, red, yellow, and green. The blue and black color cardboards have identical heights, which are approximately 1 mm lower than those of the other three colors. Figure 4(a) shows the measured sample captured in natural light. Figure 4(b) shows the dot array on the measured sample captured in dark environments. Specifically, for the images obtained at the 405-nm wavelength, the intensity of dots returned from the red cardboard is relatively weak. Similarly, the images at the 633-nm wavelength exhibit low reflection on the blue cardboard. Expectedly, all projected dots failed to accurately measure the black cardboard. Figure 4(d)–(f) display the point clouds obtained by individually processing the calculations for the three wavelengths. In the point clouds obtained from the multi-wavelength method, the missing portions in the point clouds of single wavelengths are supplied by projected light dots with other wavelengths. The multi-wavelength point cloud is shown in Figure 4(c).

Projecting a dot array with a large field of view and a high number of dots holds significant research importance. The simulation of a 99 × 99 dot array covered 120° field of view is shown in Supplementary Material Note 16. The size of the 405 nm dot array is 350 mm × 350 mm at a working distance of 100 mm, with the size of dots being 0.7 mm. There are a total of 19,395 dots in the region of 405 nm dot array, consisting of a 99 × 99 dot array for 405 nm, a 75 × 75 dot array for 532 nm, and a 63 × 63 dot array for 633 nm. To achieve a multi-wavelength dot array with equal and high density in each area, the design of the dot array can be adjusted based on different diffraction angles. Specifically, the size and gap of the projected dots with a large diffraction angle can be optimized using a reverse design method to enhance the density of dots in the edge area.

## 4 Conclusions

In summary, we designed and experimentally demonstrated a novel planar SL projection device based on metasurfaces to project a multi-wavelength dot array, including 1200 projected dots in the visible region. We introduce the PB phase to realize an identical phase distribution for different wavelengths. The experimental result is consistent with the simulation result and demonstrates our design of the nanofin. All diffraction patterns of the three wavelengths have high *LIUF* and enough *RMSE* so that all dots can be clearly distinguished in captured images. To verify the application of a multi-wavelength SL projection in 3D imaging, we processed a metasurface to project a dot array with an area of approximately 10 mm × 10 mm, including 363 projected dots. The density of the dots in the multi-wavelength method has been significantly improved, which causes a high depth accuracy and more details of the measured sample in 3D imaging result. Building upon this foundation, we can enhance the dot density of SL by increasing the number of wavelengths to achieve higher resolution. Furthermore, another notable advantage of the multi-wavelength method is offering an improved error resistance compared to single-wavelength systems. The 3D imaging system has the potential to achieve high-resolution color reconstruction in dark environments in the future. The metasurface-based multi-wavelength SL projection will enable a new generation of high-performance 3D imaging systems for a wider range of applications.

## Supplementary Material

Supplementary Material Details

Supplementary Material Details
