# Supplementary material for: Multi-wavelength structured light based on metasurfaces for 3D imaging
Source: Nanophotonics. 2024 Feb 7;13(4):477–85. doi: 10.1515/nanoph-2023-0885 (PMC11501290; doi:10.1515/nanoph-2023-0885)
Supplement: Supplementary file 1 — Supplementary Material Details [file j_nanoph-2023-0885_suppl_001.docx]

Multi-wavelength Structured Light Based on Metasurfaces for 3D Imaging

Baiying Lyu ^a,b^, Chen Chen ^b,^*, Jian Wang ^b^, Chang Li ^b^, Wei Zhang ^a,b^, Yuxiang Feng ^c^, Fei Dong ^c^, BaoShun Zhang ^a,b^, Zhongming Zeng ^a,b^, Yiqun Wang ^a,b,^*, Dongmin Wu ^a,b,^*

^a^ *School of Nano-Tech and Nano-Bionics, University of Science and Technology of China, Hefei 230026, China*

^b^ *Nanofabrication Facility, Suzhou Institute of Nano-Tech and Nano-Bionics, Chinese Academy of Sciences, Suzhou 215123, China*

^c^ *Beijing Aerospace Institute for Metrology and Measurement Technology, Beijing 100076, China*

* Corresponding authors

*Email addresses*: [cchen2017@sinano.ac.cn](mailto:cchen2017@sinano.ac.cn) (C. Chen)

*Email addresses*: [yqwang2008@sinano.ac.cn](mailto:yqwang2008@sinano.ac.cn) (Y. Wang)

*Email addresses*: [dmwu2008@sinano.ac.cn](mailto:dmwu2008@sinano.ac.cn) (D. Wu)

**Supplementary Material**

1. Simulation Results of Nanofin Structure Parameters.

After determining the period (*P*) and height (*H*) of the nanofin, a simulation is conducted to calculate the length (*L*) and width (*W*) parameters of the structure, which covers the current ice range of 30–170 nm. To accommodate the processing precision, we use the size interval of 5nm. Figure S1a and S1b show the transmittance (*T*) and polarization conversion efficiency (*PCE*) of the simulation results for the structures. Both *T* and *PCE* achieve their highest values at *L*=165 nm and *W*=65 nm.


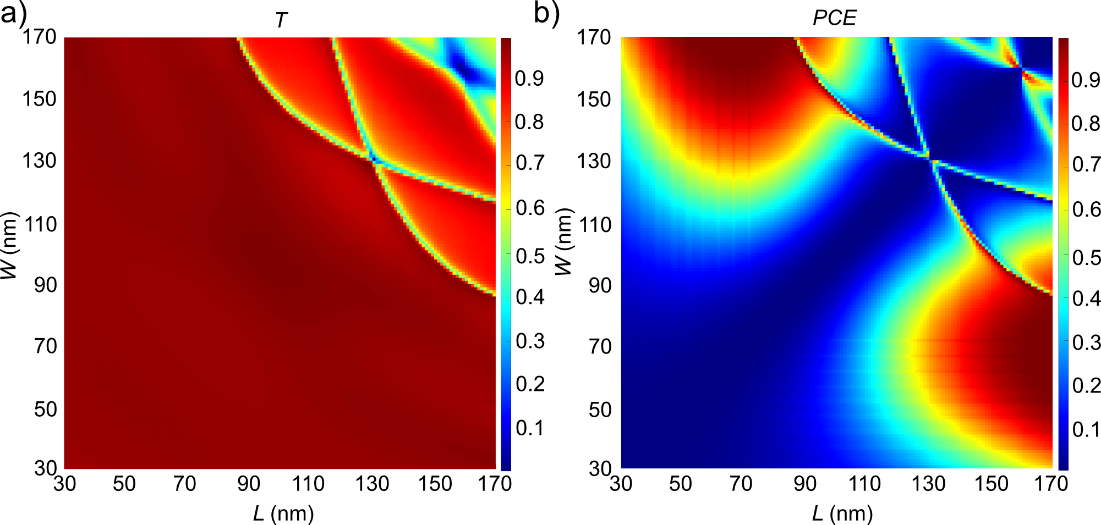


Figure S1: Simulation results of (a) *T* and (b) *PCE* concerning the scanned nanofin structure parameters *L* and *W*.

The multi-wavelength dot array improves density of dots by filling the gaps between projection dots of other wavelengths. In this study, we employed three wavelengths—405 nm, 532 nm, and 633 nm—with 405 nm being the shortest wavelength. The shorter wavelength can project a smaller dot size, which is more conducive to obtain high resolution. However, it is important to note that high resolution and a short wavelength are not entirely equivalent. Therefore, the projected dots of 532 nm and 633 nm are choosing to fill the gaps of 405 nm. The structure is specifically optimized for 405 nm to achieve a high-quality dot array.

In this paper, we have chosen parameters characterized by high *PCE* and *T* specifically at the wavelength of 405 nm. The *PCE* decreases at 532 nm and 633 nm, impacting the intensity and size of zero-order diffraction at these respective wavelengths. Moreover, considering the principles of dispersion, the diffraction angle and size of dots at 532 nm and 633 nm are larger than those at 405 nm. This facilitates the design of dot array encoding to fill the gaps of 405 nm. Additionally, the gaps at 532 nm and 633 nm are larger than those at 405 nm, preventing the overlap of projected dots and ensuring accurate calculation of their coordinates.

Optimizing the structure at the central wavelength (532 nm) has the potential to enhance *PCE* and *T* for both 532 nm and 633 nm. However, only the accuracy of the projected dots at 532 nm can be guaranteed, and there may be overlapping of the projected dots at 405 nm.

1. Complete target dot array.

The complete target dot array consists of 400 dots, including 20×20 dots, with uniform spacing among the dot. The overall size of the area is 50 mm×50 mm. Each dot pixel has the same size and an intensity value of 1, while the other pixels have an intensity value of 0.


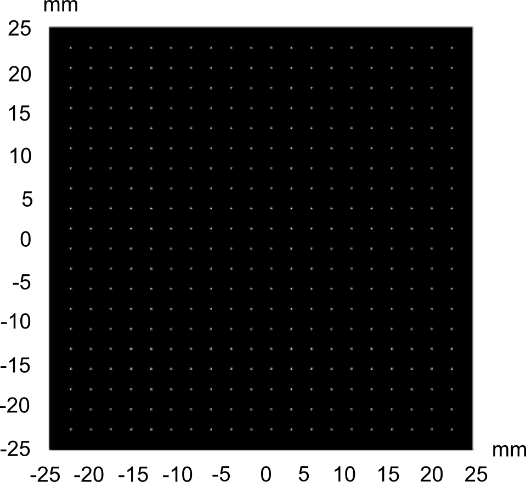


Figure S2: Schematic of the target dot array.

1. *LIUF* and *RMSE* in the iteration of G-S algorithm.

During the phase calculation of G-S algorithm, the phase distribution obtained in each iteration is calculated by diffraction simulation. Figure S3 illustrates the trends of these two values with respect to the number of iterations.


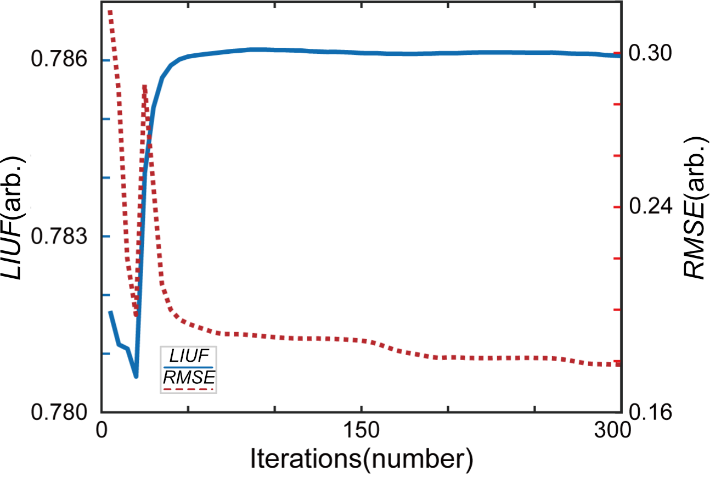


Figure S3: Variations of *LIUF* (blue curve) and *RMSE* (red curve) with increasing iteration numbers.

The *PCE* influences both the intensity and size of zero-order diffraction. In both simulations and experiments, the *LIUF* is calculated without accounting for zero-order diffraction. Following the removal of zero-order diffraction, the *LIUF* remains similar for the three wavelengths in both simulations and experiments due to their identical phase distributions.

Fresnel diffraction integral formula and *PCE* are combined to calculate the diffraction pattern of three wavelengths. Figure S4 (a)-(c) show the diffraction pattern without zero-order diffraction for three wavelengths, while Figure S4 (d)-(f) show the diffraction pattern with zero-order diffraction for the same wavelengths. It can be observed that the size of the zero-order diffraction increases as the *PCE* decreases.


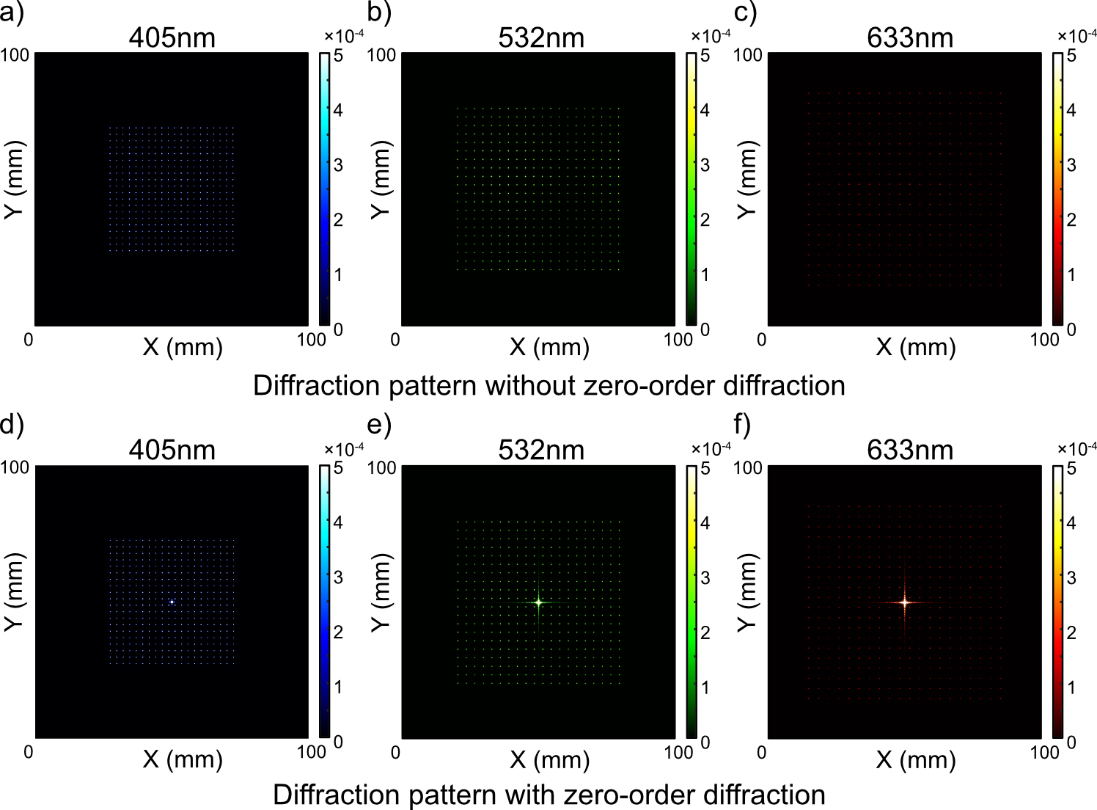


Figure S4: (a), (b), (c) Diffraction pattern without zero-order diffraction. (d), (e), (f) Diffraction pattern with zero-order diffraction.

The Table S1 displays the relative intensity of the sum of all dots, zero-order diffraction, and the normalized intensity of the diffraction pattern, respectively. Notably, as the *PCE* decreases, the intensity of zero-order diffraction increases. The *LIUF*, calculated without considering zero-order diffraction, is approximately 80% (calculated by Fresnel diffraction integral formula). When accounting for zero-order diffraction, the *LIUF* is 66.15% at 405 nm but drops to 5.57% at 633 nm.

Table S1: Relative intensity and *LIUF* data for the diffraction patterns of three wavelengths.

| Wavelengths | 405 nm | 532 nm | 633 nm |
| --- | --- | --- | --- |
| Relative intensity of sum of all dots | 0.6615 | 0.1231 | 0.0557 |
| Relative intensity of zero-order diffraction | 0.1875 | 0.6233 | 0.9349 |
| Normalized intensity of diffraction pattern | 1 | 1 | 1 |
| *LIUF* without zero-order diffraction | 81.41% | 83.30% | 85.52% |
| *LIUF* with zero-order diffraction | 66.15% | 12.31% | 5.57% |

1. Phase distribution.


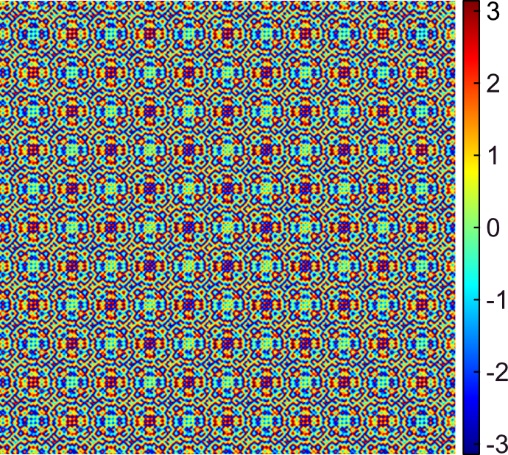


Figure S5: The phase distribution is calculated using the G-S algorithm to recover the phase of the target diffraction pattern.

1. Fabrication of the Metasurfaces

All metasurfaces consist of TiO_2_ nanofins on a fused-silica substrate. The electron beam resist was spin-coated onto a clean substrate, which was pretreated with hexamethyldisilazane. The thickness of the resist was 500 nm, which is the same height as the nanofins. A thin chromium layer (10 nm thick) is deposited to improve conductivity. Subsequently, the designed patterns were exposed by electron beam lithography (EBL). Following the development, amorphous TiO_2_ was deposited onto the resist by atomic layer deposition (ALD), with a thickness of W_max_/2 for complete filling, where W_max_ is the maximum width for the nanofins. Subsequently, reactive ion etching (RIE) was employed to remove the TiO_2_ top layer. The etching depth was as same as the deposited thickness. Finally, any residual resistance around the nanofins was removed by an oxygen plasma.

The disparity between simulated and measured values may be attributed to fabrication errors. Errors introduced during EBL lead to variations in the dimensions (length, width, and angles) of the structure, influencing corresponding phase, *PCE*, and *T*. Additionally, inconsistencies in RIE speed may result in discrepancies between actual and theoretical etching depths, impacting both structure height and optical properties.

1. Imaging setup.

In the imaging setup, the incident light is first collimated using a spatial filter composed of an objective lens and pinhole. Subsequently, a lens transforms the divergent beam into a parallel beam. Finally, a diaphragm is used to filter out the side beam. The half-waveplate and quarter-waveplate convert linearly polarized light to right-handed circularly polarized light. The diffraction patterns projected by the metasurface are then captured by a camera.

The model number of the 405 nm laser is OBIS 405nm LX 50 mW (Coherent Inc.). The model number of the 532 nm laser is AUT-FCL-532-10TX2 15 mW (Aunion Inc.). The model number of the 633 nm laser is HNL050L 5 mW (Thorlabs Inc.).


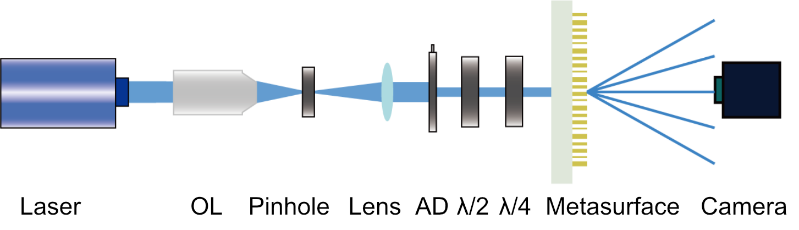


Figure S6: Schematic of the imaging setup. The incident light is 405-nm, 532-nm and 633-nm laser; OL represents the optical lens, whose magnifications is 10×; the diameter of Pinhole is 25 μm; the focus of the Lens is 50.8 mm; AD represents the aperture diaphragm, and its size is tangent to the main flap of the beam; λ/2 is a half-wave plate at 405 nm; λ/4 is a quarter-wave plate at 405 nm; Matesurface represents the processed metasurface; Camera represents the CMOS sensor.

1. Light intensity distribution of the 20×20 dot array.


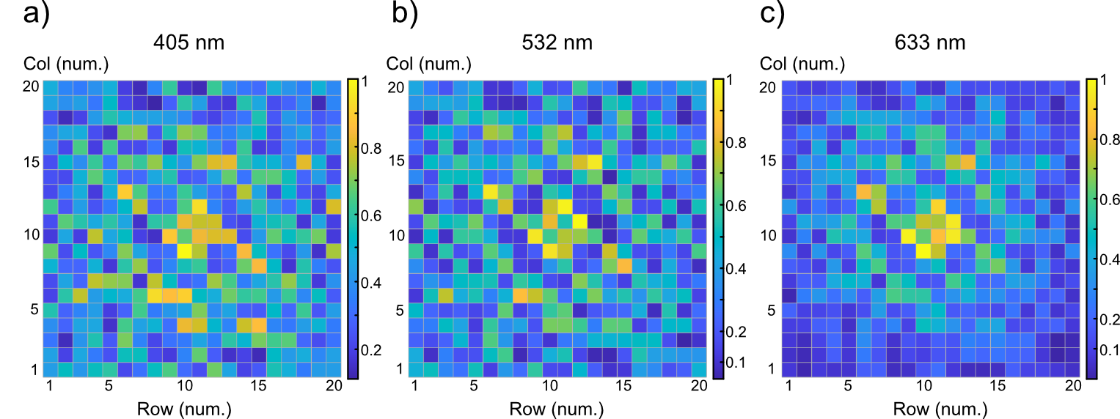


Figure S7: Light intensity distribution of the 20×20 dot array. (a), (b), (c) The light intensity of 400 projected dots with 20 points per row and 20 points per column of wavelengths of 405 nm, 532 nm, and 633 nm. The color of each square represents the normalized light intensity of the projected dots in the respective row and column.

1. Diffraction orders correspond to the diffraction angles of the 20×20 dot array.

Table S2: Diffraction orders correspond to the diffraction angles of three wavelengths.

| Diffraction order | Diffraction angle (405 nm) | Diffraction angle (532 nm) | Diffraction angle (633 nm) |
| --- | --- | --- | --- |
| 1 | 1.097° | 1.591° | 1.662° |
| 2 | 3.432° | 4.574° | 4.799° |
| 3 | 5.688° | 7.845° | 8.323° |
| 4 | 8.071° | 10.942° | 11.683° |
| 5 | 10.381° | 14.246° | 15.031° |
| 6 | 12.702° | 17.223° | 18.594° |
| 7 | 14.959° | 20.528° | 21.984° |
| 8 | 17.273° | 23.562° | 25.538° |
| 9 | 19.489° | 26.996° | 29.028° |
| 10 | 21.840° | 30.018° | 33.049° |

1. Polar map of the projected dots.

After configuring the imaging setup, the degree of circular polarization is measured of the incident lights at 405 nm, 532 nm, and 633 nm. The results are shown in Figures S9a, 9c, and 9e, respectively. Subsequently, the metasurface is put into the imaging setup. The right circular polarization degree of output lights is measured, and Figures S9b, 9d, and 9f show the results of three wavelengths. Both incident lights and output lights exhibit favorable circular polarization characteristics.

The intensity in the polar map of the output light is calculated as follows. A λ/2 wave plate of the corresponding wavelength is used in the imaging setup to adjust the beam to linearly polarized light. Subsequently, a λ/4 waveplate of the corresponding wavelength is used to further convert the linearly polarized light to right-handed circularly polarized light. To measure the polarization characteristics of the beam, a polarizer is placed behind the λ/4 waveplate and in front of the metasurface. Additionally, a camera is positioned behind the metasurface to capture the diffraction pattern.

Due to the different fields of view for each wavelength, there are six projection dots in each image taken at 405 nm and four projection dots in the images taken at 532 nm and 633 nm. After taking ten images at one angle, the polarizer is rotated by 10° and the camera captures another set of ten images. The polarizer rotates a total of 36 times. Averaging the ten images taken at the same angle, we calculate the light intensity of all projection dots in the images.


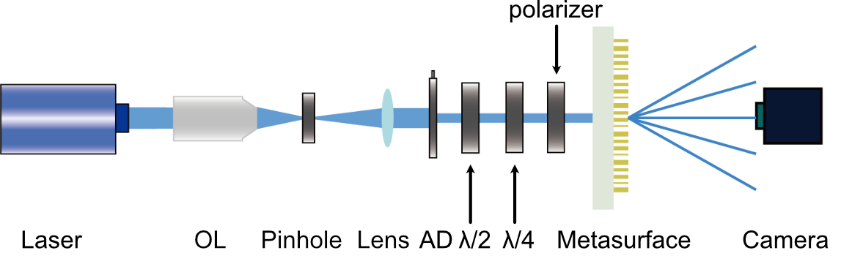


Figure S8: Schematic of the setup for polarization state characterization.


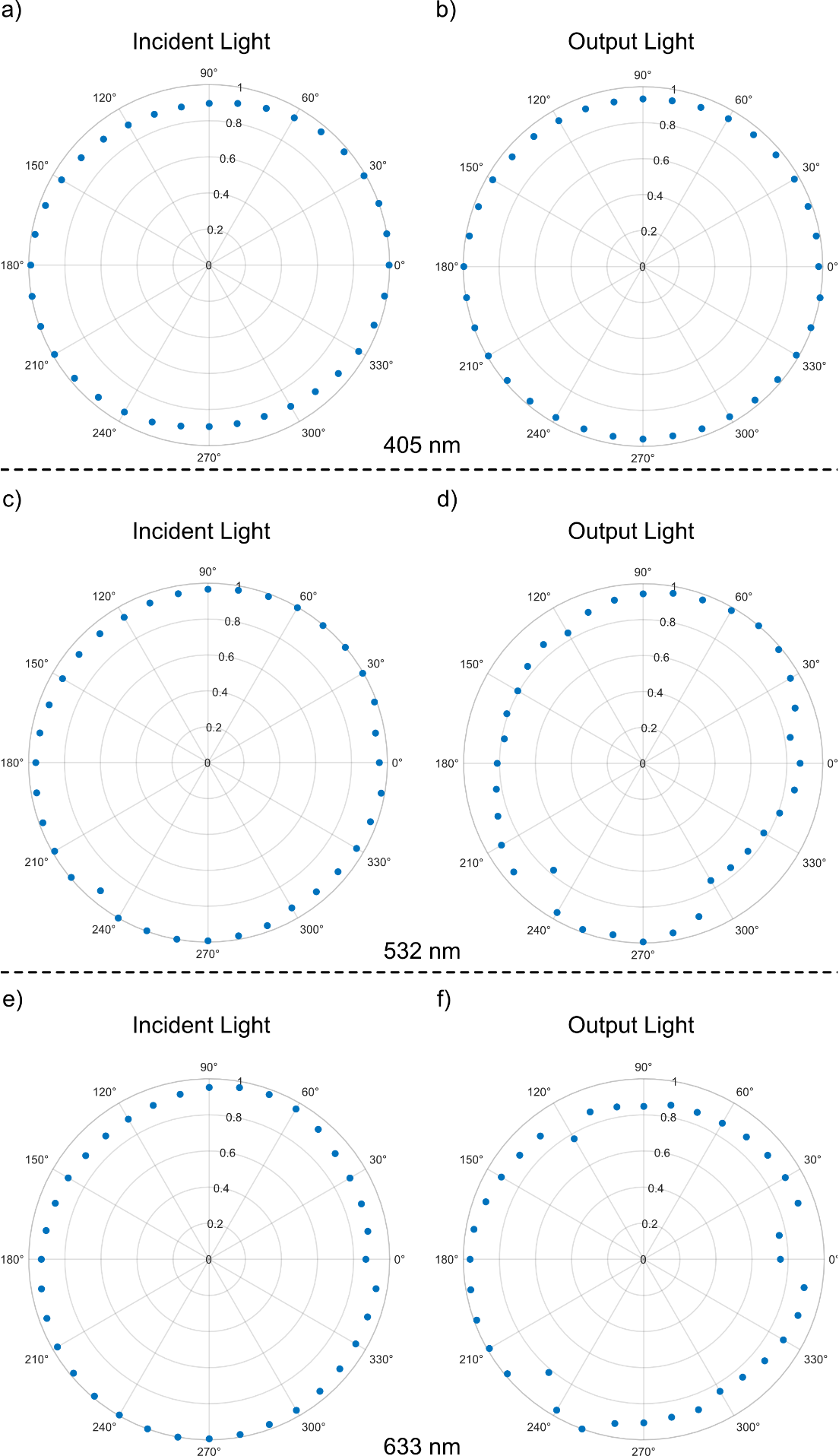


Figure S9: Polar map of incident lights and output lights of 405 nm, 532 nm, and 633 nm.

1. Checkerboard images.

Through camera calibration, the focal length and other parameters of the camera can be acquired.


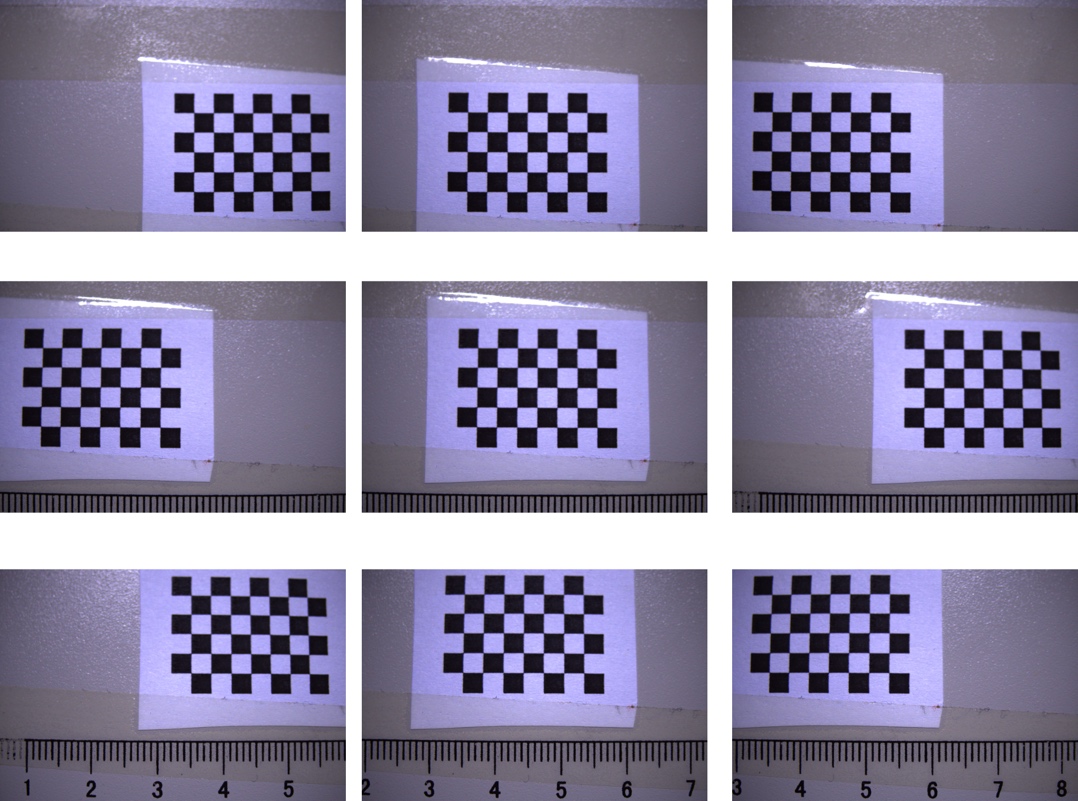


Figure S10: Checkerboard images.

1. Monocular Vision Depth Calculation Method

After acquiring camera parameter F through calibration, a monocular vision system is employed to calculate the depth of the measured object, as shown in Figure S11. The coordinate difference of the dot array is acquired by the camera on the reference plane and object. The triangular similarity principle is employed to calculate the corresponding depth change. Subsequently, the spatial coordinates of the x and y directions are calculated based on the depth change and pixel position change of the camera plane.

The baseline length is the distance between the center of the metasurface and the optical center. The lens focal length is the distance from the camera plane to the center. Z is the distance from the reference plane to the center. R, G, and B represent the intersections of the 633-, 532-, and 405-nm lights with the reference plane. P_R_, P_G_, and P_B_ are the points where the 633-, 532-, and 405-nm lights intersect with the measured object. R_1_, G_1_, and B_1_ are the points where the reflected light at 633-, 532-, and 405-nm intersects with the reference plane. R’, G’, B’ and R_1_’, G_1_’, B_1_’ are the corresponding points of R, G, B and R_1_, G_1_, B_1_ on the camera plane. Z_R_, Z_G_, and Z_B_ are the depths of P_R_, P_G_, P_B_, and Z_R_ is calculated as follows:

$Z_{R}=\frac{Z^{2}*R'R_{1}'}{F*MC-Z*R'R_{1}'}$,

where R’ R_1_’ is calculated by the difference of coordinates on the camera plane. Z_G_ and Z_B_ are similarly calculated.

The physical distance between Metasurface (M) and Reference Plane (O) is approximately 150 mm (MO). The physical distance between Metasurface (M) and Center (C) is approximately 97.5 mm (MC). The physical distance between Center (C) and Reference Plane (O) is approximately 114 mm (Z). The physical distance between Camera Plane (O’) and Center (C) is approximately 10.272 mm (F).


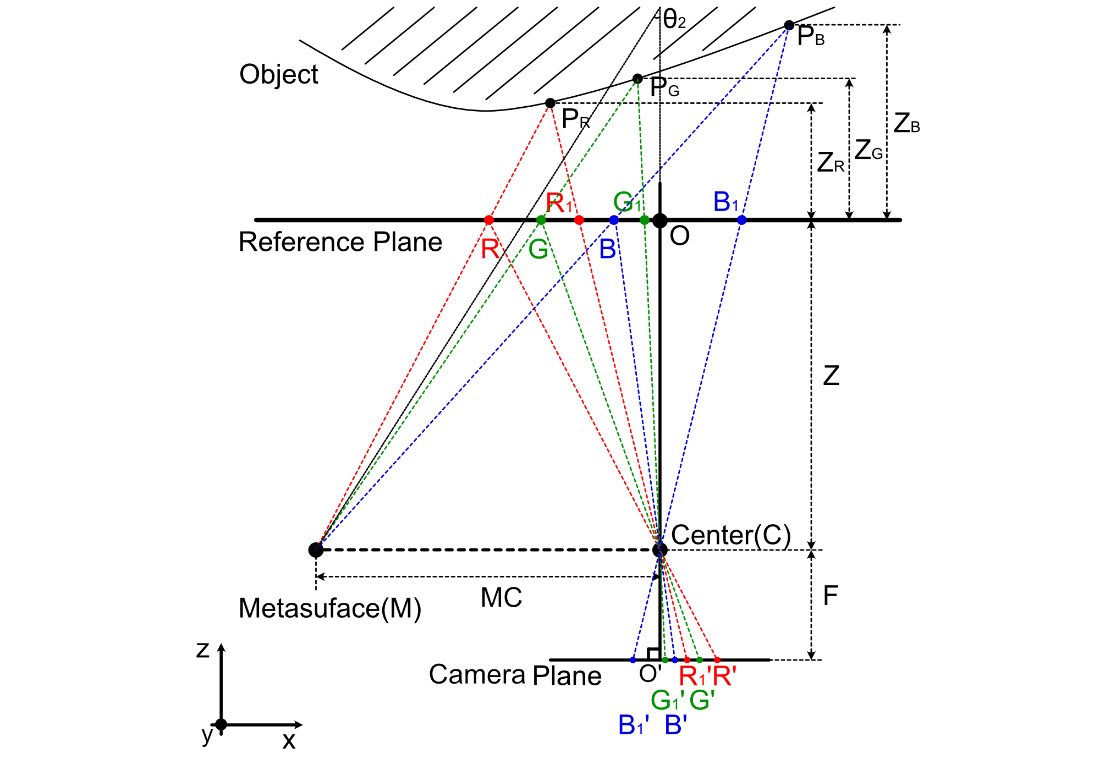


Figure S11: Schematic of the monocular vision depth measurement.

The model number of the camera is MER-630-60U3C-L (DAHENG Imaging Inc.). The sensor is 1/1.8" IMX178 Rolling shutter CMOS (Sony Inc.). Figure S13 shows the spectral curve of the camera. The model number of the lens is UC Series Fixed Focal Length Lens 12mm (Edmundoptics Inc.).


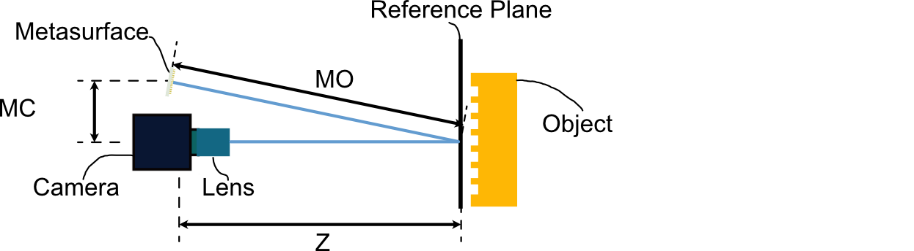


Figure S12: Schematic of the monocular vision system.


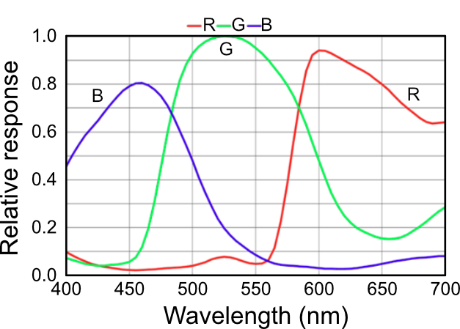


Figure S13: Spectral curve of the camera.

1. Point clouds of Areas 1–3 are calculated using the multi-wavelength SL projection.


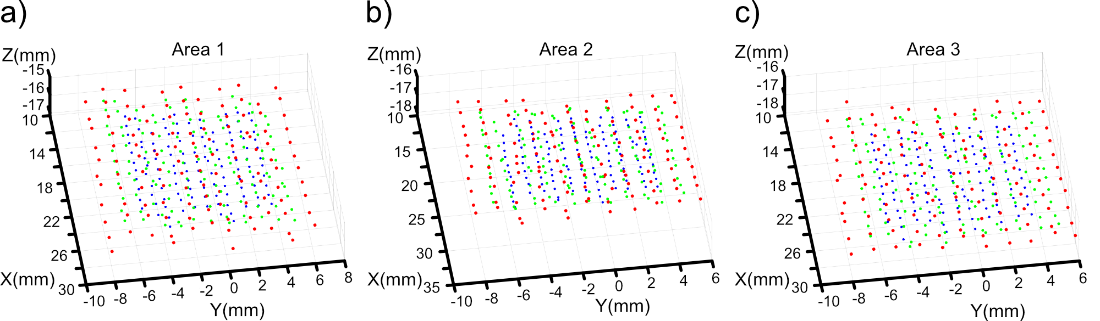


Figure S14: Point clouds of (a) Area 1, (b) Area 2, and (c) Area 3 calculated using the multi-wavelength SL projection.

1. Measured object 2 with steps of varied depths is made using 3D printing.

In Figure S15, we have incorporated a hole with increased depth at the location corresponding to the zero-order diffraction. This modification aims to mitigate the impact of light reflection from zero-order diffraction, particularly in the context of coordinate calculations for dots at the center of the projection. The presence of zero-order diffraction can potentially disrupt the accuracy of these calculations.


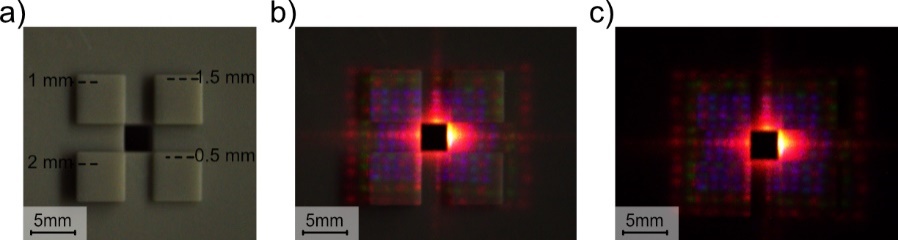


Figure S15: (a) Image of the measured object 2. (b)-(c) Image of the multi-wavelength dot array on the measured object 2 of natural environment (b) and dark environment (c).

Four curves for the measured object 2 are selected, as shown in the Figure S16. The height values of each step in the four curves were calculated individually. The standard and average values are presented in the Table S3.

Table S3: The average and curves values of Step 1to Step 4.

|  | Average (mm) | | Curves 1 (mm) | Curves 2 (mm) | Curves 3 (mm) | Curves 4 (mm) |
| --- | --- | --- | --- | --- | --- | --- |
| Step 1 (0.5 mm) | | 0.5964 | 0.5780 | 0.6010 | 0.6122 | 0.5945 |
| Step 2 (1 mm) | | 0.9098 | 0.8869 | 0.9289 | 0.8869 | 0.8978 |
| Step 3 (1.5 mm) | | 1.4575 | 1.2964 | 1.5443 | 1.4375 | 1.5516 |
| Step 4 (2 mm) | | 2.0125 | 2.0211 | 2.0528 | 1.9215 | 2.0547 |


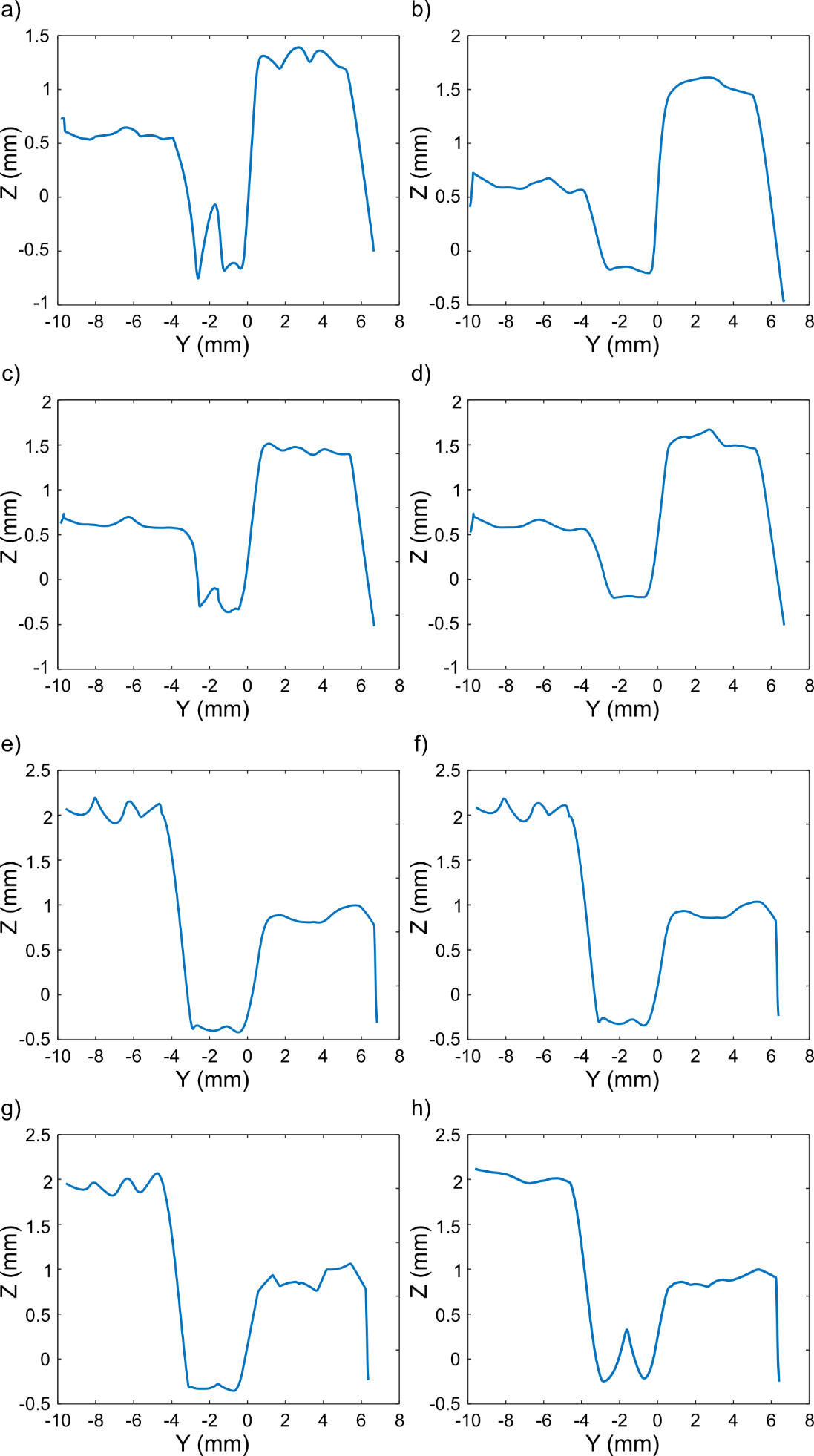


Figure S16: Curves for the measured object 2. (a)-(d) The 4 curves of Step 1 (0.5 mm) and 3 (1.5 mm). (e)-(h) The 4 curves of Step 2 (1 mm) and 4 (2 mm).

1. Detailed width data of the grooves.

Table S4: Width data of multi-wavelength and three single-wavelength imaging.

|  | Theoretical width | Multi-wavelengths | 405 nm | 532 nm | 633 nm |
| --- | --- | --- | --- | --- | --- |
| Groove 6 | 4.10 mm | 4.33 mm | 4.34 mm | 3.88 mm | 3.95 mm |
| Groove 5 | 3.70 mm | 3.81 mm | Null | Null | 4.10 mm |
| Groove 4 | 2.90 mm | 2.98 mm | Null | 3.56 mm | 2.60 mm |
| Groove 3 | 2.50 mm | 2.50 mm | 2.65 mm | 2.30 mm | 2.53 mm |
| Groove 2 | 2.10 mm | 2.18 mm | 1.71 mm | 2.15 mm | 3.51 mm |
| Groove 1 | 1.70 mm | 1.68 mm | 1.73 mm | 2.43 mm | 3.38 mm |

1. Point clouds of Area 3 are calculated using three single-wavelength SL projections.


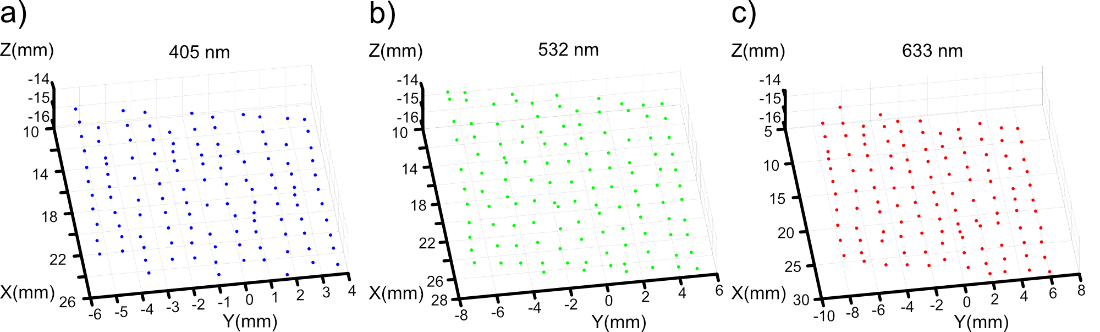


Figure S17: Point clouds of Area 3 calculated using the (a) 405-nm, (b) 532-nm, and (c) 633-nm SL projection.

1. Simulation of large field of view dot array.

For the large field of view and a large number of projected dots, we designed a 99×99 dot array, with the size of dots being 0.7 mm and the gap between dots being 2.8 mm. The size of the dot array is approximately 350 mm, and the working distance is 100 mm. The field of view of 405 nm dot array is approximately 120 degrees. The result is shown in the Figure S18. There is a 75×75 dot array for the 532 nm wavelength in the region of the 405 nm dot array, while there is a 63×63 dot array for the 633 nm wavelength in the same region. There are a total of 19,395 dots in the region of 405 nm dot array.


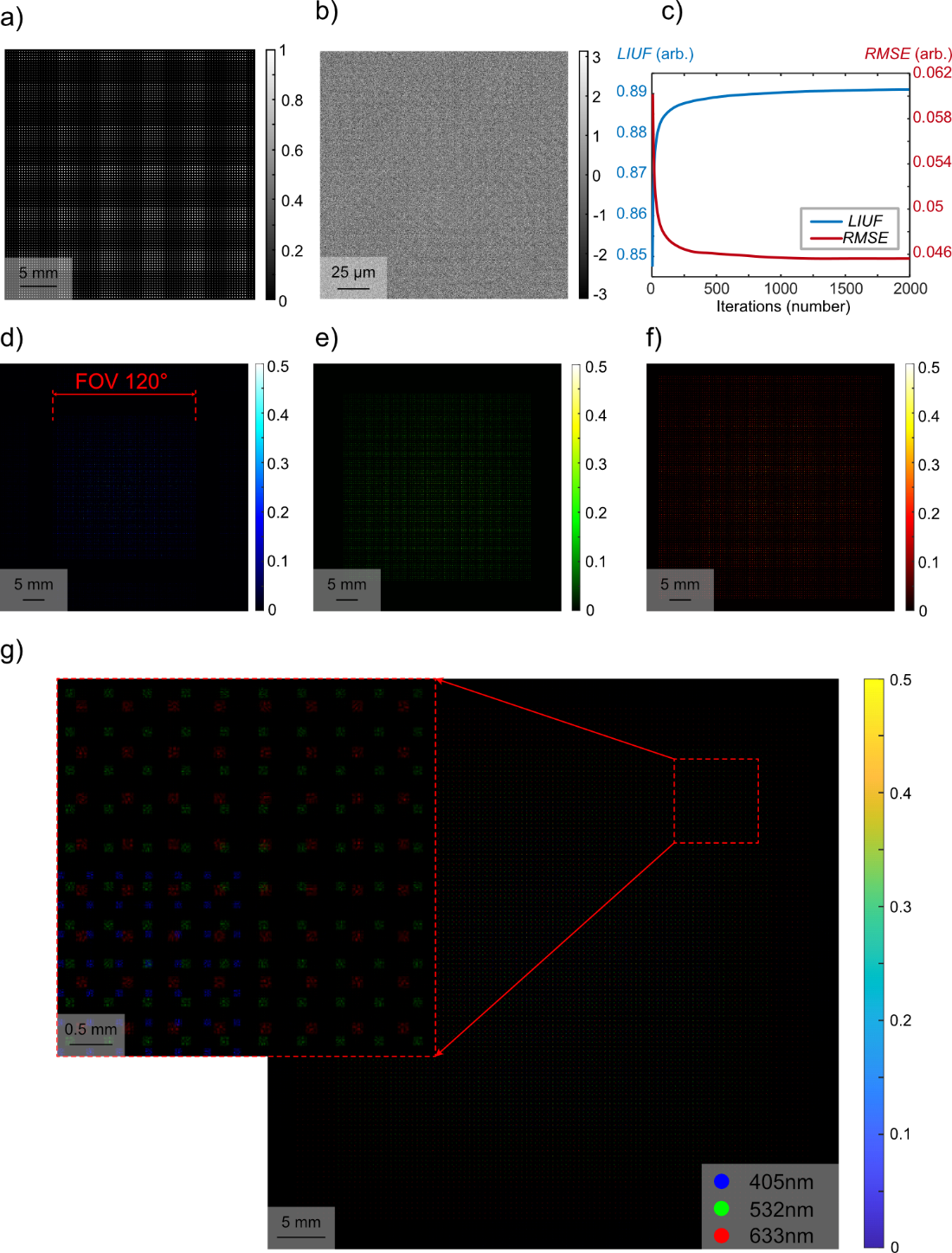


Figure S18: Simulation of large field of view multi-wavelength dot array. (a) Schematic of the target of 99×99 dot array, with the size of dots being 0.7 mm and the gap between dots being 2.8 mm. The size of the dot array is approximately 350 mm. (b) The phase distribution is calculated using the G-S algorithm to recover the phase of the target 99×99 dot array. (c) Variations of *LIUF* (blue curve) and *RMSE* (red curve) with increasing iteration numbers. (d)-(f) Simulation of the dot array calculated using Fresnel diffraction integral formula at 405 nm, 532 nm, and 633nm, respectively. The field of view of 405 nm dot array is 120°. (g) Simulation of the multi-wavelength dot arrays calculated using Fresnel diffraction integral formula. The red dotted box area represents the edge of the 405 nm dot array.
